# Application of MinION sequencing as a tool for the rapid detection and characterization of *Listeria monocytogenes* in smoked salmon

**DOI:** 10.3389/fmicb.2022.931810

**Published:** 2022-08-10

**Authors:** Sarah Azinheiro, Foteini Roumani, Ana Costa-Ribeiro, Marta Prado, Alejandro Garrido-Maestu

**Affiliations:** ^1^Food Quality and Safety Research Group, International Iberian Nanotechnology Laboratory, Braga, Portugal; ^2^Department of Analytical Chemistry, Nutrition and Food Science, Faculty of Veterinary Science, University of Santiago de Compostela, Lugo, Spain; ^3^Department of Biochemistry, Genetics and Immunology, University of Vigo, Vigo, Spain

**Keywords:** long-read sequencing, MinION, *Listeria monocytogenes*, serotyping, ready-to-eat, smoked salmon, antimicrobial resistance

## Abstract

Microbial pathogens may be present in different types of foods, and hence the development of novel methods to assure consumers' safeness is of great interest. Molecular methods are known to provide sensitive and rapid results; however, they are typically targeted approaches. In recent years, the advent of non-targeted approaches based on next-generation sequencing (NGS) has emerged as a rational way to proceed. This technology allows for the detection of several pathogens simultaneously. Furthermore, with the same set of data, it is possible to characterize the microorganisms in terms of serotype, virulence, and/ or resistance genes, among other molecular features. In the current study, a novel method for the detection of *Listeria monocytogenes* based on the “quasimetagenomics” approach was developed. Different enrichment media and immunomagnetic separation (IMS) strategies were compared to determine the best approach in terms of *L. monocytogenes* sequences generated from smoked salmon samples. Finally, the data generated were analyzed with a user-friendly workflow that simultaneously provided the species identification, serotype, and antimicrobial resistance genes. The new method was thoroughly evaluated against a culture-based approach, using smoked salmon inoculated with *L. monocytogenes* as the matrix of choice. The sequencing method reached a very low limit of detection (LOD50, 1.2 CFU/ 25 g) along with high diagnostic sensitivity and specificity (100%), and a perfect correlation with the culture-based method (Cohen's *k* = 1.00). Overall, the proposed method overcomes all the major limitations reported for the implementation of NGS as a routine food testing technology and paves the way for future developments taking its advantage into consideration.

## Introduction

Food contamination, particularly with microbial pathogens, remains a major health issue in developed and developing countries. In 2020, in Europe, a generalized drop in the number of outbreaks and human cases was reported. This observation was probably associated with two major events: the COVID pandemic and the withdrawal of the UK from the reporting system. However, hundreds of thousands of cases of foodborne infections were still reported, such as 120,000 cases of campylobacteriosis and 50,000 cases of salmonellosis, among others (EFSA and ECDC, 2021).

Currently, in addition to the presence of specific pathogens, it is of utmost importance to also monitor for bacteria that carry antimicrobial resistance genes (ARGs). It was estimated that, in the European Union, more than 670,000 infections every year are associated with antimicrobial-resistant microorganisms (ARMs), and these are directly responsible for 33,000 deaths (ECDC, 2022). Many of the ARMs of highest relevance worldwide, such as *E. coli* or *S. aureus*, may be found in foods; furthermore, among them, some are well-known human pathogens, such as *Salmonella* spp. or *Shigella* spp. (WHO, 2014).

The standard protocol followed in food microbiology is to perform individual assays to culture and isolate a target pathogen. If successful, subsequent analysis, such as serotyping and genetic characterization attending to virulence and ARG, may be performed. This approach results in lengthy workflows that are not fully compatible with the intensive production systems which are ongoing nowadays. For some time now, many types of methodologies have been reported to improve, or directly overcome, the limitations of culture-based methods. Many novel approaches implement different types of biosensors (Kang et al., 2010; Yoon and Kim, 2012; Queirós et al., 2014; Dao et al., 2018; Umesha and Manukumar, 2018). However, the most widely accepted alternative methods rely on nucleic acid amplification, which includes techniques such as loop-mediated isothermal amplification (LAMP) or recombinase polymerase amplification (RPA; Notomi et al., 2000; Piepenburg et al., 2006), but undoubtedly the gold standard of these type of techniques is polymerase chain reaction (PCR) and real-time PCR (qPCR). A myriad of methods have been published implementing these two techniques for targeting different foodborne pathogens, and some of them have even undergone interlaboratory validation studies (D'Agostino et al., 2004; Malorny et al., 2004; Delibato et al., 2014; Gianfranceschi et al., 2014; Cheng et al., 2015). However, all these sensors, techniques, and methods present one common limitation, that is, they are all targeted approaches as they always seek for one specific microorganism, thus there is always a risk of missing other pathogens which may be present in the sample under study. Alternatively to the targeted methods, non-targeted ones, such as those based on DNA sequencing, may be a better way to proceed (Cocolin et al., 2018). These techniques are typically applied to pure microbial cultures in order to typify them and in metagenomic studies to characterize the populations present in the sample (González-Escalona et al., 2019; Jagadeesan et al., 2019; Chen et al., 2020). However, if a laboratory is interested in the direct detection of the microbial pathogens present in a food sample, the workflows must be adapted for this particular application, and it is in this context that major gaps exist, as only a handful of studies have been reported (Katz et al., 2017; Forghani et al., 2020; Ottesen et al., 2020; Townsend et al., 2020; Commichaux et al., 2021). Most of these studies take advantage of the so-called “quasimetagenomics” approach described by Hyeon et al. (2018), which, in brief, is based on a primary sample enrichment followed by immunomagnetic separation (IMS) to concentrate on one specific pathogen, whole-genome amplification (WGA) to increase its DNA concentration, and finally identification and characterization of the pathogen by DNA sequencing. This approach allows for the detection and characterization of the pathogen of interest obtained from a complex matrix at the genomic level without the need for isolating and purifying the bacteria. Alternative to this approach, Azinheiro et al. reported a “semi-targeted” method to allow for the simultaneous detection of different foodborne pathogens which were further characterized at the serotype level (Azinheiro et al., 2021). These authors performed parallel enrichments which were combined for DNA extraction and analysis. Briefly, the “quasimetagenomics” approach allowed for the detection and acquiring in-depth characterization of one pathogen, while the “semi-targeted” approach was capable of simultaneously recovering several pathogens which were subtyped at the serotype level.

The goal of the current study was to develop a novel method based on the “quasimetagenomics” approach for the detection and characterization of *Listeria monocytogenes* in smoked salmon samples for its application as a routine testing method. To this end, and considering the bottlenecks and limitations of routine testing laboratories, the efforts of the study focused on the following points:

1. Provide a simple enrichment procedure.
2. Optimize the IMS step.
3. Evaluate the suitability of an automatic sequencing data analysis workflow.
4. Reduce costs associated with the sequencing platform.

## Materials and methods

### Bacterial strains and preparation of fresh cultures for spiking experiments

For the development and initial evaluation of the methodology, *L. monocytogenes* WDCM 00021 serotype 4b, acquired from the Spanish Type Culture Collection, was used as the reference strain (World Data Center for Microorganisms). This strain was selected based on the fact that this is one of the reference strains that has been indicated by the ISO standards for *L. monocytogenes* analyses (ISO, 2003a, 2009, 2017). In addition to this strain, four other strains isolated from mollusks, chicken, and chestnuts were also included, and additional details are provided in Table 1.

**Table 1:** *L. monocytogenes* strain list.

**Source**	**Origin**	**ORF 2819**	**ORF 2110**	***lmo0737***	***lmo1118***	***hly***	**Serogroup**	Serotype*
Spinal fluid	CECT (WDCM 00021)	+	+	-	-	+	IV	**4b**, 4d, 4e
Mollusk	Spain	+	-	-	-	+	III	**1/2b**, 3b
Chicken	Portugal	+	-	-	-	+	III	**1/2b**, 3b
Chestnut	Spain	-	-	+	-	+	I	**1/2a**, 3a
Chicken	Spain	-	-	+	-	+	I	**1/2a**, 3a

* The serotype provided for strain WDCM 00021 was confirmed to be 4b by the Spanish Type Culture Collection (CECT); for the other strains, two serotypes are given based on the serogrouping results and interpretation provided by Vitullo et al., and highlighted in bold indicates the most probable one provided (Vitullo et al., 2013).

Fresh cultures of each strain were prepared by resuspending one single colony in 4 mL of nutrient broth (NB, Biokar diagnostics S.A., France) and incubated overnight at 37°C. These fresh cultures were serially diluted 100-fold times for salmon spiking experiments, and in parallel plated on tryptic soy yeast extract agar (TSYEA, Biokar diagnostics S.A., France). The plates were incubated at 37°C overnight to obtain a reference value of viable microorganisms.

The spiking experiment was performed by distributing 100 μL, or 10 μL, of the corresponding dilution prepared as detailed above over 25 g of salmon. Subsequently, the broth for the primary enrichment was added (the details regarding primary enrichment optimization are given in the following section), and the mixture was homogenized. This was a method to mimic a real scenario wherein the bacteria are dispersed on the surface of the food and must undergo the “stress” generated by the homogenization step.

### DNA extraction

#### Extraction from pure cultures for molecular serogrouping characterization

The DNA for molecular serogrouping (see Materials and methods Section Molecular serogrouping) was extracted by bacterial thermal lysis. To this end, the protocol described by Azinheiro et al. was selected (Azinheiro et al., 2020b). Briefly, 1 mL of a pure *L. monocytogenes* culture was centrifuged at 16,000 × *g* for 2 min, the supernatant was discarded, the pellet was rinsed with 1 mL of TE (10 mM Tris-HCl and 1 mM EDTA, pH 8.0), and centrifuged again. The supernatant was discarded, and the pellet was resuspended in 200 μL of TE. The suspension was heated at 99°C for 10 min with constant agitation at 1,400 rpm in a dry bath (Thermomixer comfort, Eppendorf AG, Germany). Finally, the heat-treated suspension was centrifuged for 2 min at 16,000 × *g* and 4°C. The supernatant was transferred to a new tube and stored at −20°C.

#### Extraction from food samples

Two milliliters of the secondary enrichment (details are provided in the following section) was taken and centrifuged at 16,000 × *g* for 2 min, the supernatant was discarded, the pellet was resuspended in 1 mL of 1X TE, and centrifuged again as previously indicated. The clean bacterial pellets were used for DNA extraction with the DNeasy PowerSoil Pro Kit (Qiagen, Barcelona, Spain) implementing the preliminary step recommended by the kit for “difficult to lyse cells”. In continuation, the standard procedure was followed, and DNA was eluted in 30 μL, and stored at −20°C.

### Molecular serogrouping

All the strains of *L. monocytogenes* tested in the current study were serogrouped following the method described by Vitullo et al. (2013). Briefly, the experiments consisted of multiplex qPCR reactions that were performed in a final reaction volume of 25 μL with the following components: 12.5 μL of NZYSupreme qPCR Probe Master Mix 2X (NZYTech, Lisbon, Portugal) 300 nM primers and 100 nM probes for *lmo0737*, ORF2819, and ORF2110, while for *lmo1118*, 1,000 nM primers and 200 nM probe were added. A total of 5 μL of template DNA was loaded, and the remaining volume was filled with sterile MilliQ water. The thermal profile selected included a hot-start step at 95°C for 5 min, followed by 40 cycles of dissociation at 94°C for 5 s, and annealing-extension at 60°C for 30 s. These experiments were performed in a QuantStudio 5 Real-Time PCR System, with the QuantStudio™ Design and Analysis Software v1.4.3 (Applied Biosystems™). The sequences of all the primers and probes are provided in Table 2.

**Table 2:** Primer and probe sequences.

**Oligonucleotide**	**Target**	**Sequence 5' → 3'**	**Modification**	**References**
ORF2819-F	ORF2819	ATC ACT AAA GCC TCC CAT TGA G	-	Vitullo et al., 2013
ORF2819-R		GGA AGA TTT CCA CGC AAT ACT C	-	
ORF2819-P		CTC GTA AGA T//CG ATA TAC GTC ATG GCA GTT TCC	FAM™/ZEN™/IB^®^FQ	
lmo0737-F	*lmo0737*	GCA TCT TGT TTA GCA AGT GGA TC	-	
lmo0737-R		GAG CAC GGA AGT TGC TAG GT	-	
lmo0737-P		CCA ACA CTT TCT CAT CAA TAC CAT CTT CCC	TEX™615/ IB^®^RQ	
ORF2110-F	ORF2110	CAC TAA TCT CAT CGA CTA TAA ACT C	-	
ORF2110-R		TGC ACA AGC AGC AGA GGA AG	-	
ORF2110-P		TCT CCG TCA T//TT GTT ACC GTT TCC CCA AC	HEX™/ZEN™/IB^®^FQ	
lmo1118-F	*lmo1118*	CTT AGT ATT CCA GGA TTT AAG ACC	-	
lmo1118-R		CCA AAG AAC CAA ATT GAT CGA ATC	-	
lmo1118-P		CCT TTA TCT TCT CCT GAG TGT ATA CGC CTC	TYE™665/IB^®^RQ	
hly-P3F	*hly*	CGC AAC AAA CTG AAG CAA AGG A	-	Roumani et al., 2021
hly-P3R		CGA TTG GCG TCT TAG GAC TTG C	-	
hly-P3P		CAT GGC ACC//ACC AGC ATC TCC G	FAM™/ZEN™/IB^®^FQ	
IAC-P	IAC	AGT GGC GGT//GAC ACT GTT GAC CT	YY™/ZEN™/IB^®^FQ	Garrido-Maestu et al., 2018
IAC-DNA		^a^GGA TTA CCC TAG AGT GGC GGT GAC ACT GTT GAC CTT CTA TTA CCT C	-	

a The sequence is flanked by the hly primers as the IAC is competitive, and so amplified with the same set of primers as the target microorganism.

### MinION optimization

**Figure 1:**
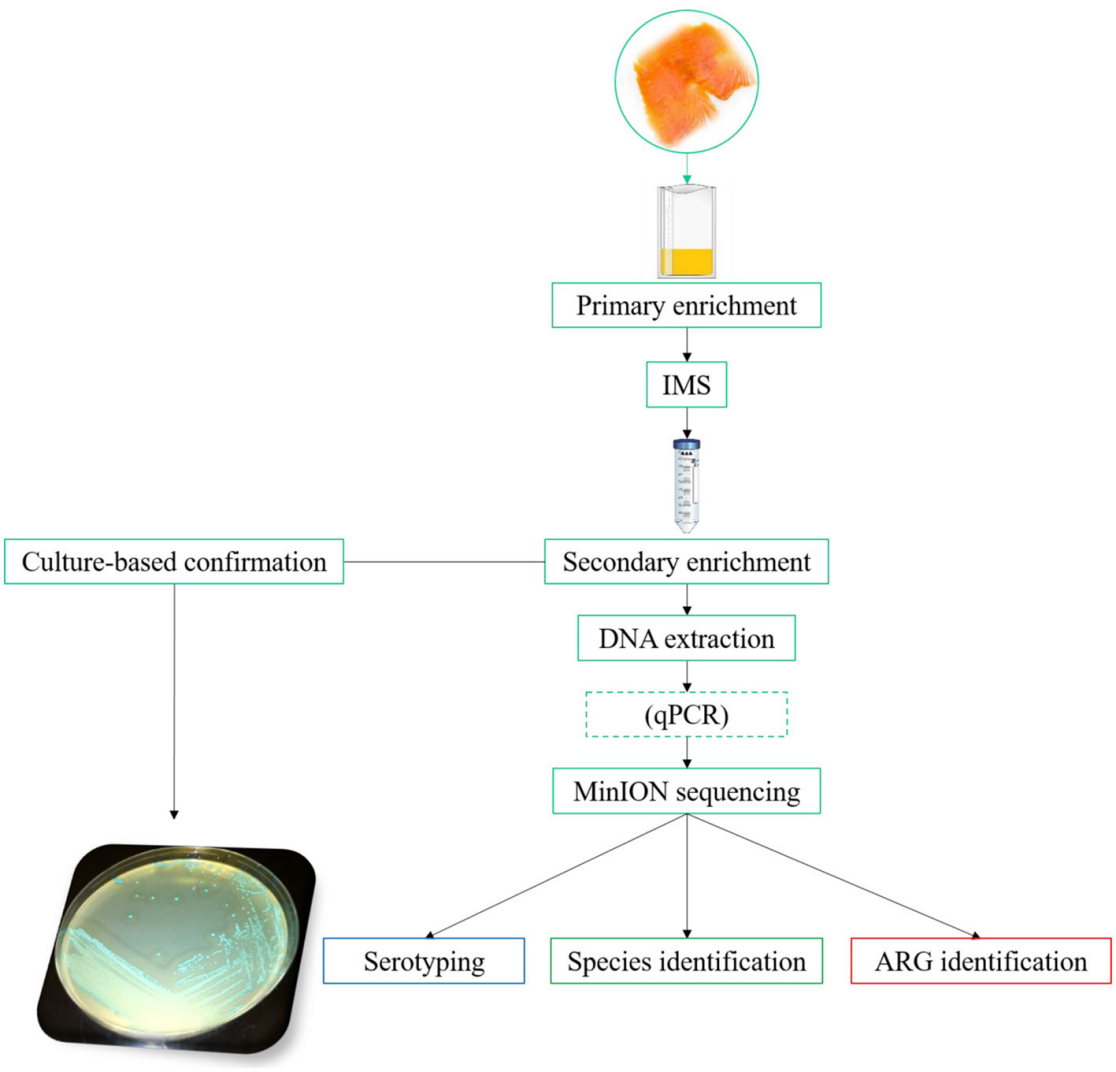
General workflow. For the “primary enrichment,” HF and ONE broth *Listeria* were compared. In the “IMS” step, the commercial Dynabeads^®^ anti-*Listeria* was compared with MNP functionalized with a mAb. The dashed box for “qPCR” indicates that this step is optional. The “antimicrobial resistance” pipeline, available from EPI2ME, for the MinION data analysis implements WIMP and CARD for species/ serotype identification and antimicrobial resistance gene detection, respectively.

The general workflow followed in the method developed is summarized in Figure 1. In the optimization of the MinION methodology, two different steps were evaluated in order to increase the concentration of *L. monocytogenes*, and at the same time, reduce the interfering microorganisms. These steps were as follows: the selection of an appropriate primary enrichment broth and the selection of the most suitable IMS protocol. The evaluation of these steps was based on the results obtained by qPCR, in terms of Cq value, and MinION sequencing, considering the percentage of reads of *L. monocytogenes* and the number of non-*L. monocytogenes* reads obtained. All the samples used for this step were spiked with 10-100 CFU of a fresh culture of *L. monocytogenes* prepared as described in Materials and Methods section Bacterial strains and preparation of fresh cultures for spiking experiments.

#### Primary enrichment broth evaluation

Two different broths were tested for the primary enrichment, namely, Half Fraser broth (HF, Biokar diagnostics S.A., France) and ONE Broth-*Listeria* (ONE, OXOID, Hampshire, UK). For the initial assessment of the performance of the different enrichment media, the IMS was performed with the magnetic beads functionalized monoclonal antibody (mAb) against *L. monocytogenes*.

The study that aimed to compare the primary enrichment methods was performed in triplicate. To this end, 25 g of smoked salmon was inoculated with 10–100 CFUs of a fresh culture of *L. monocytogenes* prepared as indicated in Materials and methods Section Bacterial strains and preparation of fresh cultures for spiking experiments, and mixed with 225 mL of the corresponding broth. The matrix was homogenized in a Stomacher 400 Circulator (Seward Limited, West Sussex, UK) for 30 s and incubated at 30°C for 24 h. After the primary enrichment, 2 mL was taken for IMS using magnetic beads functionalized with mAbs as detailed below. The concentrated bacteria were resuspended in 100 μL of PBT (0.1 M sodium phosphate buffer with 0.05% Tween^®^20, pH 7.4) and added to 10 mL of Full Fraser broth (FF, Biokar diagnostics S.A., France) for secondary enrichment and incubated at 37°C overnight. The following day, 2 mL was taken for DNA extraction as detailed in Materials and Methods section Extraction from food samples, and analyzed by MinION as specified below. Additionally, a loopful was streaked on COMPASS *Listeria* Agar (COMPASS, Biokar diagnostics S.A., France) to confirm growth on positive samples. The plates were incubated for 48 h at 37°C and screened for typical colonies.

#### Immunomagnetic separation (IMS) comparison

Two different strategies were considered. One approach was based on using Dynabeads^®^ anti-*Listeria* (Applied Biosystems™, Foster City, CA, USA) following the standard protocol, which is briefly described below. The second strategy was based on the functionalization of AbraMag^®^ Magnetic Nanospheres (MNPs, average size of 500 nm) coated with protein A (Abraxis Inc., Warminster, PA, USA) with the mAb MAB8953 (Abnova, Taipei City, Taiwan). Garrido-Maestu et al. reported optimal performance in terms of species specificity and capture efficiency, when implementing this Ab for an IMS-qPCR method (Garrido-Maestu et al., 2020). The details about the MNP functionalization protocol is provided in the supporting information.

The comparison was performed by analyzing three independent smoked salmon samples spiked with *L. monocytogenes* WDCM 00021, as detailed in Materials and methods Section Primary enrichment broth evaluation. The IMS protocol followed for both types of magnetic particles included taking 2 mL of the primary enrichment in HF, adding 40 μL of the corresponding magnetic particles to it, and incubating the mixture at room temperature in a rocker for 15 min. This step was followed by the concentration of the beads in a magnetic particle concentrator (Dynal^®^MPC, Invitrogen, Carlsbad, CA, USA) for 3 min. Subsequently, the supernatant was carefully removed, the beads were rinsed with 1 mL of PBT, and separated again after 3 min. Finally, the PBT was removed, the beads were resuspended in 100 μL of PBT, and added to 10 mL of FF. The FF tubes were incubated at 37°C overnight.

#### MinION analysis

##### Library preparation

The libraries were prepared following the standard protocol of the Rapid Barcoding Kit (SQK-RBK004), adapted for Flongle flow cells (FLO-FLG001). The maximum number of samples loaded per run was limited to four.

##### Data analysis

The base calling was performed in real-time, and the sequence analysis was performed with the workflow “Fastq Antimicrobial Resistance,” which integrates “What's In My Pot” (WIMP) for species identification followed by ARG identification using the Comprehensive Antibiotic Resistance Database (CARD; Alcock et al., 2020). Both analysis pipelines were accessed through EPI2ME™ (https://epi2me.nanoporetech.com). WIMP utilizes “centrifuge” *kmer*-based read identification (Charalampous et al., 2019). The “minimum abundance cutoff” was set at 1.0% for the species level identification.

The results obtained in terms of read abundance of *L. monocytogenes* and the number of different species identified, along with the target bacterium, were used for the selection of the most appropriate primary enrichment broth and IMS procedure.

### Multiplex qPCR pre-screening

The multiplex qPCR assay developed by Roumani et al. was introduced for sample pre-screening prior to MinION sequencing (Roumani et al., 2021). This assay targets the *hly* gene of *L. monocytogenes* and also includes a competitive internal amplification control, see Table 2 for detailed sequences. The reaction mixture consisted of 200 nM of forward and reverse primers, 150 nM of the probe, 100 nM of IAC probe, 1,000 copies of IAC DNA, 10 μL of TaqMan^®^Fast Advanced Master Mix (Applied Biosystems™, Foster City, CA, USA), 3 μL of template, and the remaining volume up to 20 μL was filled with sterile MilliQ water. The “Fast” run mode was selected, and the thermal profile consisted of an initial UDG treatment at 50°C for 2 min, hot-start activation at 95°C for 2 min, 40 cycles of dissociation at 95°C for 1 s, and annealing-extension at 63° C for 20 s. All the qPCR experiments were performed in a Quant Studio 5 Real-Time PCR System, with the QuantStudio™ Design and Analysis Software v1.4.3 (Applied Biosystems™).

### Methodology evaluation

The final method included a primary enrichment of 24 h in HF, followed by IMS with MNP functionalized with the mAbs (~20 min), and secondary overnight enrichment (~14 h) in FF. This was followed by DNA extraction (~1 h), library preparation (~45 min), and MinION sequencing (4 h) with real-time base calling (a rapid pre-screening qPCR may be done before sequencing). For comparison purposes, the secondary enrichment was plated on COMPASS as detailed in Materials and methods Section Primary enrichment broth evaluation. The evaluation of the methodology was performed based on the analysis of spiked samples to determine the limit of detection (LOD) and the overall performance in terms of relative sensitivity (SE), specificity (SP), accuracy (AC), and Cohen's kappa (k). The specific procedures followed for the determination of these parameters are detailed in the following sections.

#### Determination of the LOD

The calculation of the LOD was based on the function POD described by Wilrich and Wilrich, which estimates the probability of detecting the target microorganism at a given concentration (Wilrich and Wilrich, 2009). In order to determine this parameter, four sets of four salmon samples were spiked with decreasing concentrations of *L. monocytogenes* WDCM 00021 with the goal of reaching a concentration where both positive and negative samples were obtained (the spiking procedure is detailed in the Materials and methods Section Bacterial strains and preparation of fresh cultures for spiking experiments). These data were inputted into the model to statistically calculate the so-called PODLOD.

#### Performance assessment

Once the PODLOD was determined, additional samples were spiked above this value with different concentrations of the reference strain to calculate the performance parameters SE, SP, AC, and k. All the samples were classified to be positive or negative agreements (PA/ NA) if the results obtained by the qPCR/ MinION method matched those of the culture-based procedure, and positive or negative deviations (PD/ ND) if the results did not match those of the reference. Once classified, the data were used to calculate the mentioned parameters following the formulae described in the NordVal regulation (NordVal, 2017).

### Statistical analysis

The qPCR Cq values, as well as the % of reads, were statistically compared with the Mann–Whitney test. These analyses were performed with GraphPad Prism version 8 for Windows (GraphPad Software, La Jolla California USA, www.graphpad.com).

## Results

### MinION optimization

#### Primary enrichment broth selection

**Figure 2:**
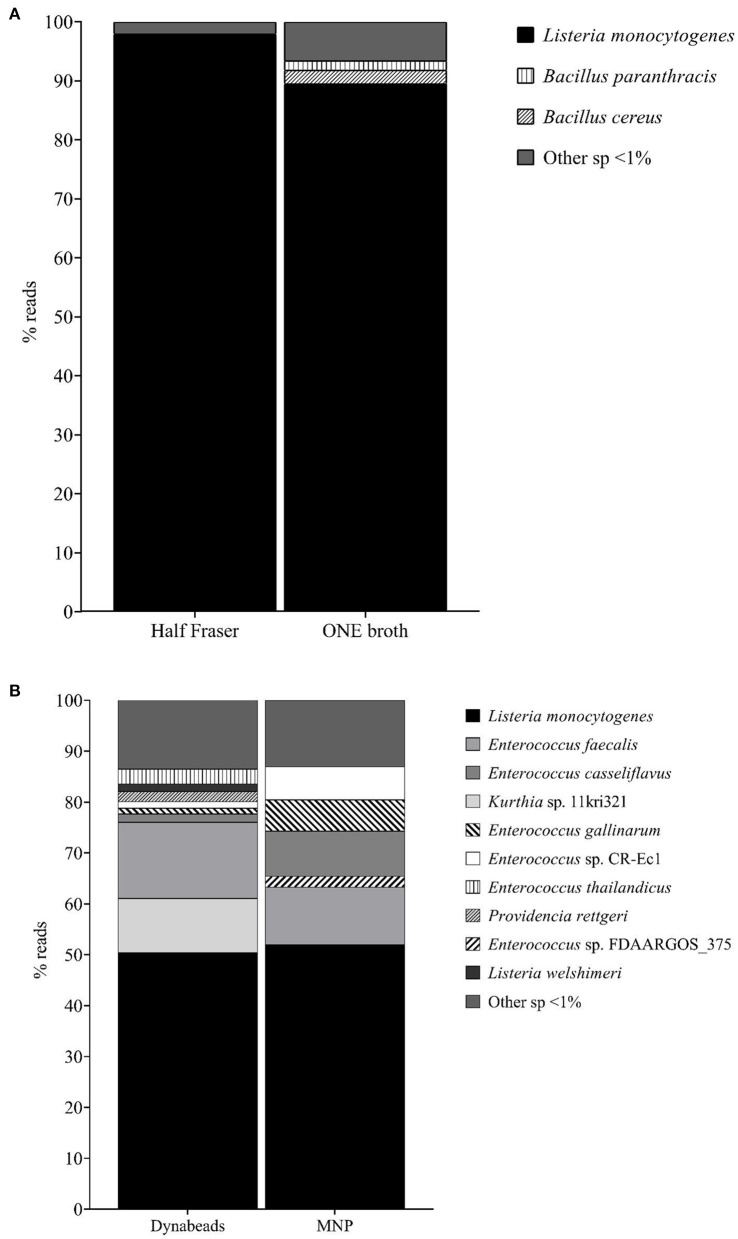
**(A)** Percentage reads obtained by WIMP in the evaluation of the two “primary enrichment” broths, HF and ONE. **(B)** Percentage reads obtained by WIMP in the evaluation of the two “IMS” approaches, Dynabeads^®^ anti-*Listeria* and MNP with the mAb.

The effect of the primary enrichment broth on the final method was based on the MinION sequencing results. It was observed that, in general, the overall number of reads classified was higher when ONE was used as the primary enrichment broth in comparison to HF. However, the percentage of *L. monocytogenes* identified was lower (88.6 vs. 96.5%; see Figure 2A). This was associated with the fact that a higher number of non-target microorganisms were also increased, as it can be observed in Figure 2A. Considering these findings, HF was selected to conduct the following steps of evaluation and the selection of the most appropriate magnetic beads.

#### Immunomagnetic separation (IMS) comparison

The qPCR analysis revealed a lower average Cq value when using Dynabeads^®^ compared to the MNPs (13.93 ± 4.50 vs. 16.35 ± 1.77). These differences were not statistically significant; however, they provided an indication that a higher concentration of *L. monocytogenes* cells, and hence DNA, was being recovered with the Dynabeads^®^ compared to the MNP. When analyzing the sequencing data, a similar observation to that of the primary enrichment evaluation was made. Overall, a higher percentage of reads were identified as *L. monocytogenes* when the Dynabeads^®^ were used (50.1 vs. 49.4%), but again the differences were not statistically significant.

Similar to what was observed in the evaluation of the enrichment broths, when the Dynabeads^®^ were applied for the IMS step, a higher number of non-target microorganisms were identified along with *L. monocytogenes*, as it can be observed in Figure 2B. For this reason, the MNPs functionalized with the mAbs were selected for the final identification.

### Methodology evaluation

#### Determination of the LOD

**Figure 3:**
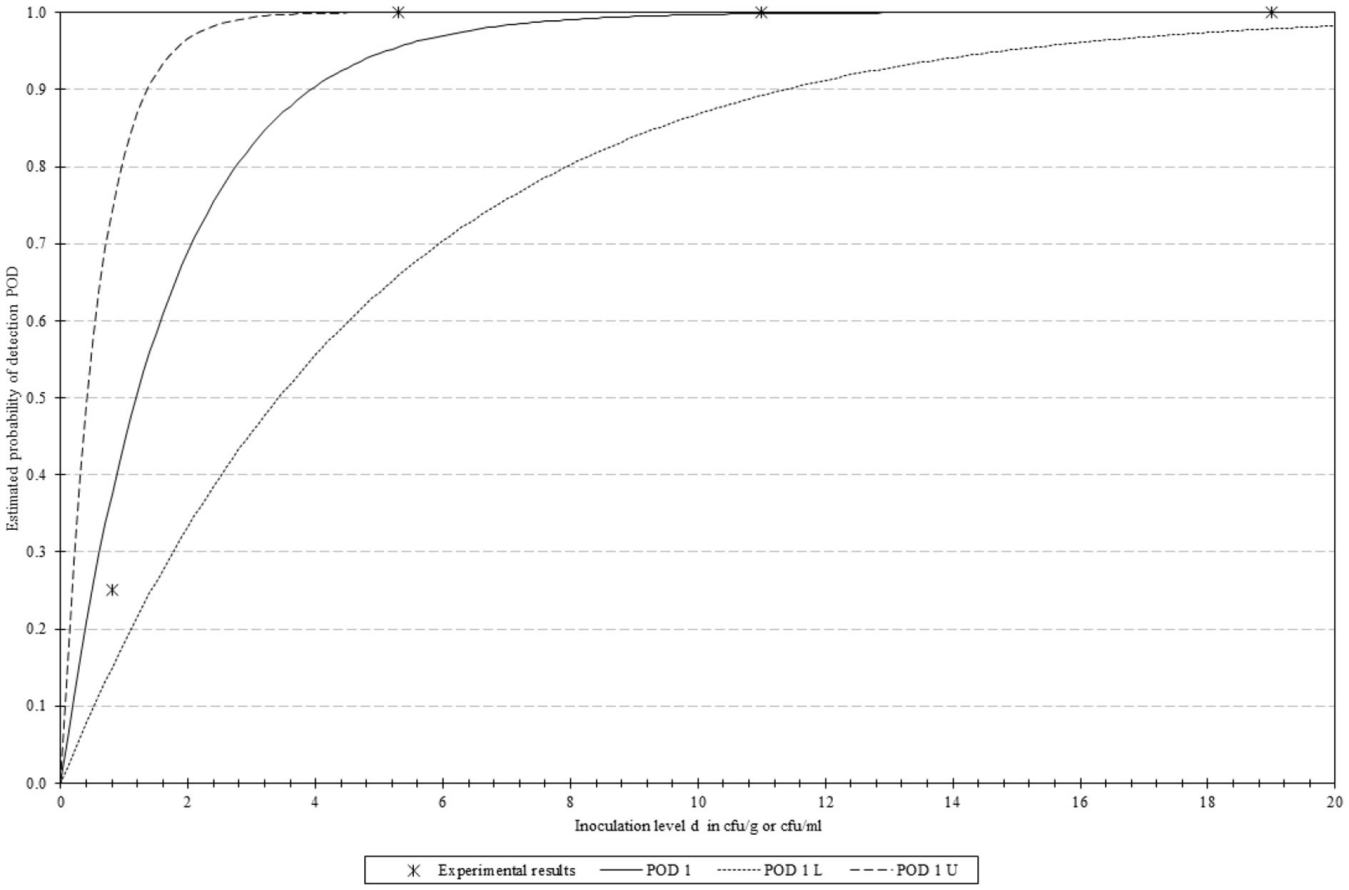
Graphical representation of the PODLOD results. “POD 1(d)” represents the Probability Of Detection, with “POD 1 L” and “POD 1 U” indicating the lower and upper limits with 95% confidence.

A total of 16 samples were spiked in groups of four, with each group containing decreasing concentrations (19.0–0.8 CFUs/ 25 g) of the sample. The mathematical model determined that the LOD50 and LOD95 values of the method were 1.2 and 5.1 CFU/ 25 g. These results, along with the corresponding confidence intervals, are graphically depicted in Figure 3.

#### Performance assessment

Considering the LOD50, a total of 32 samples were analyzed (5 negative and 27 positive) and inoculated with different concentrations in the range of 10–100 CFU/ 25 g. A total of five different *L. monocytogenes* strains, belonging to three different serogroups, were selected for the spiking of the positive samples. These included the reference strain WDCM 00021 (serogroup IV), one isolate was obtained from chicken and the other from chestnuts (serogroup I), and another two isolates obtained from chicken and mollusk (serogroup III). It should be noted that the two chicken isolates were isolated from different brands, one in Spain and the other one in Portugal. The serogroup classification was performed following the qPCR procedure described by Vitullo et al. (2013), and the details are presented in Supplementary Figures 1–3.

The evaluation of QC data of the different sequencing runs indicated that the average fragment size was 1,883 ± 974 bp, with an average quality of 10.73 ± 0.67. It was not surprising that the SD of the fragment size was relatively large due to the fact that the “raw” whole genome was being sequenced, and not a specific fragment with a defined size.

*Listeria monocytogenes* was correctly identified in all the samples, regardless of the spiking level or the serogroup selected. In addition, all five negative samples showed negative results. Considering these findings, the method obtained a value of 100% for the SE, SP, AC, NPV, and PPV, in addition to the value of 1.00 for Cohen's k [interpreted as “very good concordance” according to Altman (1991)].

### Serogrouping capacity of the workflow

Out of the 27 positive samples spiked, 19 were inoculated with serogroup IV, 5 with serogroup I, and 3 with serogroup III. Overall, the proposed method was capable of directly discriminating among the various serogroups tested in the current study, regardless of the origin of the bacterium. More specifically, the percentage of reads was correctly allocated when serogroup IV was chosen for spiking, which ranged from 52.4 to 75.7%; for serogroup I, the range of percentage was 1.6–86.2%; and the percentage corresponding to serogroup III was between 13.0 and 45.7%.

### ARM identification

The selection of the “Fastq Antimicrobial Resistance” workflow allowed to obtain, in one single analysis, information on the presence of the target microorganism (*L. monocytogenes*) to perform a simple preliminary characterization in terms of serotype, both in the WIMP analysis, and at the same time to seek for genes involved in antibiotic resistance by screening in the CARD.

The selected workflow identified a total of five different genes involved in antimicrobial resistance in the strains tested. The reference strain (WDCM 000021, serogroup IV) presented the genes *norB, msrA*, and *mprF*. The isolate from chestnut, belonging to serogroup I, harbors the highest number of resistance genes, that is, *norB, mprF, msrA, mepA*, and *FosX*. Lastly, the isolate from serogroup III, isolated from chicken in Portugal, presented the genes *norB, mprF*, and *msrA*. No resistance genes were identified in any of the experiments for the isolates obtained from chicken from Spain or for the one coming from the mollusk, belonging to serogroups I and III, respectively.

### Multiplex qPCR pre-screening

The qPCR was introduced in the method as an optional step. Following the described procedure, the overall run only takes 20 min, and considering that all positive samples had Cq values lower than 20 (the average Cq value for all the samples included in the current study was 14.79 ± 2.36, ~10 min), they can be moved into the sequencing workflow without additional delay as the same DNA extract can be used.

All the samples with a positive qPCR were confirmed by MinION sequencing and classical microbiology. Likewise, all samples negative by qPCR were also negative by MinION and by the culture-based approach, thus confirming the utility of the qPCR as a rapid pre-screening tool. The implementation of this step allows for reducing the number of samples to be analyzed by MinION sequencing.

## Discussion

Even though many different molecular methods, and detection platforms, have been developed in recent years (El Sheikha et al., 2018; El Sheikha, 2021; Garrido-Maestu and Prado, 2022), when it comes to the detection of foodborne pathogens, which typically are present in foods at very low concentrations, some kind of sample treatment is always needed (Brehm Stecher et al., 2009). The classical way to proceed is to culture the microorganisms in a suitable broth. However, when bacteria with different physiological characteristics and properties need to be identified, this task can be challenging as the media tend to have inhibitors to limit the growth of non-target microorganisms. A way to overcome this limitation is to perform parallel cultures for all the bacteria of interest, but we would then face a second limitation related to the fact that detection platforms can only identify a limited number of targets per run. A way to solve these problems may be by the implementation of non-targeted detection strategies, such as those based on DNA sequencing. Following this approach, one could not only identify any pathogen potentially present in our food sample, but also reduce the time required for subsequent characterization, as the original DNA data can be used to identify antimicrobial resistance genes and/ or virulence factors, among other genetic markers of interest.

Up to date, very few studies have been published with regard to the implementation of DNA sequencing as a simultaneous detection and characterization tool for food samples, and even less have performed a detailed evaluation of its analytical performance (Hyeon et al., 2018; Forghani et al., 2020; Ottesen et al., 2020; Azinheiro et al., 2021; Commichaux et al., 2021; Wagner et al., 2021). This may be in part due to the complexity involved in the recovery of the microorganisms to reach an acceptable concentration for the sequencing analysis. This limitation was partially overcome by Hyeon et al. with their “quasimetagenomics” approach focused on *Salmonella* spp. by implementing an IMS step and the whole-genome amplification of the DNA prior to the sequencing step (Hyeon et al., 2018). In the current study, we evaluated a similar approach for the detection of *L. monocytogenes*.

The selection of a proper primary enrichment broth was initially assessed. To this end, Half Fraser broth, which is indicated in the ISO standard for the detection of *L. monocytogenes* (ISO, 2004), and ONE Broth *Listeria*, a commercial medium that was reported to outperform HF (Azinheiro et al., 2020a), were compared. It was observed that the total number of reads of *L. monocytogenes* obtained after enrichment in ONE was higher when compared to that obtained in HF; however, the percentage was lower. Al-Zeyara et al. evaluated these two broths in spiked food samples and also observed a higher concentration of *L. monocytogenes* in ONE compared to HF. The authors attributed this observation, among other reasons, to the natural microflora present in the various foods (Al-Zeyara et al., 2011). In addition to this, we speculate that these results could be associated with a higher selectivity of HF, as it was observed that the percentage of not only *L. monocytogenes* but also the non-target bacteria, naturally present in the smoked salmon, identified by sequencing was lower. This hypothesis was further supported by the fact that, according to Azinheiro et al., the growth of *L. monocytogenes* is slower in HF when compared to ONE (Azinheiro et al., 2020a). This observation can be explained by the fact that HF contains 3 g/ L of lithium chloride that inhibits the growth of certain Gram-positive bacteria, and previous studies have already reported that this antimicrobial agent also shows some effect on the growth of *L. monocytogenes* (Jacobsen, 1999). The lower number of reads obtained in HF was not regarded as a major limitation of the methodology, as the overall percentage was higher than that obtained for ONE, and this would not be the final sample to be analyzed, but the starting point of the method. Furthermore, the fact that it allowed for the better removal of natural microflora present in the food was considered as an added value, as it would allow for better enrichment of *L. monocytogenes* for the downstream steps of the method.

In the second step, two different types of magnetic nanoparticles were compared to determine which one produced the best results in order to be further implemented in the IMS step. These were the commercial Dynabeads^®^ anti-*Listeria*, which were reported to also capture *Listeria* spp., and MNP functionalized with a monoclonal antibody against *L. monocytogenes* previously described (Garrido-Maestu et al., 2020), which have the added value of being smaller and the corresponding advantages that a reduced size provides (Yang et al., 2007). Similarly to what was observed in the primary enrichment broths, the number of reads corresponding to *L. monocytogenes* after the IMS treatment with the Dynabeads^®^ was higher than that of the MNP; however, the percentage of reads of *L. monocytogenes* after IMS-MNP was higher, and also the reads from other microorganisms were lower (less non-*L. monocytogenes* species identified with a lower number of reads). This observation was attributed to the fact that the Dynabeads^®^ are coated with polyclonal antibodies which can bind to other *Listeria* spp., and even potentially to other non-related species (Vytrasová et al., 2005), while the functionalization of the MNP with the mAb improved the specificity of the IMS treatment.

The optimization previously described allowed for the removal of a great deal of the interfering bacteria, thus increasing the number of *L. monocytogenes* transferred to the secondary enrichment step. Implementing a secondary enrichment step brings the overall turnaround time to ~44 h considering the whole method (primary and secondary enrichments, IMS, DNA extraction, DNA barcoding, and sequencing); however, it allows to avoid the need for a WGA step included in the protocol of Hyeon et al. (2018), while providing a high concentration of pure *L. monocytogenes* even in a complex food matrix. Performing the sequencing experiments with a high DNA concentration has been reported to be a critical step for a successful outcome (Maguire et al., 2021).

The results obtained in the current study indicated that it was possible to detect *L. monocytogenes* by MinION sequencing with high diagnostic sensitivity, specificity, and accuracy in spiked smoked salmon samples. All the analyzed performance parameters obtained values of 100%, along with a Cohen's k of 1.00. The last parameter, Cohen's k, provides an overall idea about how good is the match between the two methodologies, and in this particular case, the value is interpreted as “almost in complete concordance” as described by Altman (1991). Additionally, it is worth noting that a very low LOD was also achieved (1.2 and 5.1 CFU/ 25 g for the LOD50 and LOD95, respectively), making the method suitable for the intended application. These results are in agreement with those previously reported for other molecular assays targeting *L. monocytogenes* and implementing other technologies, such as PCR/ qPCR, LAMP, RPA, and/or other techniques (Cimaglia et al., 2016; Garrido-Maestu et al., 2018; Kim and Oh, 2021; Roumani et al., 2021). As previously commented, not many studies have focused on the application of this technology for the detection of microbial pathogens, thus it was not possible to perform a comparison with other methods applying the same detection platform. It must be kept in mind that sequencing platforms are commonly used for the identification and characterization of microbial isolates once the bacteria have been isolated and purified from foods.

In addition to the good results reported, from the author's point of view, the method presents another three interesting features. The first one relies on the capacity to simultaneously perform preliminary characterization of the isolates, thus saving time in subsequent steps, as will be discussed below. The second relies on the extremely low likelihood of false-positive results associated with the detection of DNA from dead bacteria due to the implementation of a two-step enrichment protocol as detailed by D'Agostino et al. (2015). This has been regarded as one of the major limitations of nucleic acid-based methods (Elmerdahl Olsen, 2000; Kobayashi et al., 2009), and the current protocol is capable of overcoming it. The third advantage relies on the fact that the enrichment procedure followed was the one detailed in the ISO standard for *L. monocytogenes*; considering that according to the international protocols for the validation of alternative methods, such as NordVal or ISO (ISO, 2003b; NordVal, 2017), positive results obtained by an alternative method must be confirmed following the standard one, thus our protocol allows to avoid additional delays in the confirmation process, as the initial enrichment steps have already been performed and the laboratory may proceed with the selective plating (in our culture-based approach, it was observed that only typical colonies of *L. monocytogenes* grew on the selective agar plates due to the efficient removal of non-target bacteria as detailed above).

The major goal of the current study was to provide an easy and economically affordable sequencing method to be used as a routine analysis tool. This goal was successfully achieved by implementing a two-step standard enrichment process, a well-known DNA extraction procedure, and an automatic sequencing data analysis workflow. However, the authors are aware that even with the multiplexing capacity and implementation of the Flongle, some small laboratories may prefer to select specific samples to be sequenced to further reduce costs. For this reason, it was decided to include an optional pre-screening step by performing multiplex qPCR. According to the results obtained, an excellent correlation between the proposed qPCR and the MinION sequencing was obtained, as all the samples positive by qPCR were also positive by MinION sequencing. The implementation of this step does not represent a significant delay in the sequencing results, as by running it in “Fast format,” positive results may be obtained in as little as 14–19 cycles of amplification regardless of the initial bacterial concentration. If the thermal profile selected is kept in mind, positive results can be detected in as little as 15 min (considering UDG treatment and hot-start activation), and negative ones can be discarded in ~20 min. This rapid pre-screening is possible due to the high concentration of *L. monocytogenes* obtained at the end of the secondary enrichment, after the optimized IMS step. The qPCR assay implemented for pre-screening includes a competitive IAC to reduce the hands-on work and less pipetting of reagents, while increasing the confidence in the results by assuring lack of reaction inhibition (Rip and Gouws, 2009). It is worth noting that no qPCR inhibition was observed in any of the samples analyzed, most likely due to the two-step enrichment method which reduces the food debris transferred to the final sample, along with the implementation of the DNeasy PowerSoil Pro kit, which has been reported to be highly efficient in removing PCR inhibitors, such as humic acid, from a wide variety of samples (Pearman et al., 2020; Magnani, 2021; Cantu et al., 2022).

To further explore the capabilities of the proposed method, it was attempted to confirm its serotyping capacity. To this end, a panel of *L. monocytogenes* from the laboratory's collection was serogrouped by qPCR. One characteristic feature of this serogrouping technique is the fact that each group gathers different serotypes; however, there is always one that is more prevalent than the others, which is the more likely serotype (Doumith et al., 2004). Overall, it was possible to differentiate the various serogroups tested, regardless of the origin of the isolate. However, some difficulties were observed in the correct identification of serogroup I, in the samples inoculated with the isolate obtained from chestnuts (most probable serotype, 1/2a). It was observed that one sample spiked with this serogroup revealed serotypes 3b and 4b as the most likely ones. This particular sample had a low number of subclassified reads (i.e., 184). This issue can be easily solved by either increasing the sequencing time or switching to a regular flow cell, as a higher number of reads can be obtained at the same time due to the higher number of pores (2,048 vs. 504).

Regarding the identification of resistance genes, the workflow presented performs the gene identification based on the CARD database (McArthur et al., 2013), and allowed for the identification of several antimicrobial resistance genes in the isolates tested. Three of the strains included in the study had the genes *norB*, which confers resistance to quinolones; *msrA*, which encodes for the methionine sulfoxide reductase and repairs proteins inactivated by oxidation; and the *mprF*, gene that encodes for the phosphatidylglycerol lysyltransferase and produces lysyl phosphatidylglycerol, a compound involved in the resistance to cationic antimicrobial peptides. In addition, the isolate obtained from chestnuts, belonging to serogroup I, was also bearing the genes *mepA* and *FosX*, which encode for the multidrug export protein, and the fosfomycin resistance protein, respectively. Similarly, a previous study focusing on the sequencing of a multidrug-resistant 1/2a *L. monocytogenes* strain from Brazil identified *norB, msrA*, and *mepA*, along with others (Haubert et al., 2018). The gene *FosX* has been extensively reported in the isolates of *L. monocytogenes* worldwide (Wilson et al., 2018; Yu et al., 2021).

As a final remark, the authors would like to comment that alternatively to the workflow described herein, there are a few public, cloud-based servers that can also be used to analyze the reads recovered from the MinION. These include Galaxy (Jalili et al., 2021, https://usegalaxy.org) and Nanopore Galaxy (https://nanopore.usegalaxy.eu/), along with the one from the Center for Genomic Epidemiology (https://www.genomicepidemiology.org/services/). All these servers provide different tools which are freely available to conduct a similar analysis to those reported in this study, along with some others; however, these require additional training, and the results of the sequencing runs have to be realized, while the presented works have the advantage that the results are analyzed in real-time without any additional delay.

## Conclusion

In the current study, a MinION long-read sequencing method for the detection and characterization of *L. monocytogenes* was successfully developed. The methodology begins with a comprehensive, ISO-compatible, two-step enrichment protocol, which was combined with an IMS *L. monocytogenes* concentration/purification step taking advantage of a mAb, instead of the polyclonal antibodies commercially used. In addition, by implementing automatic genomic data analysis, the species identification, along with the serotype and antimicrobial resistance, can be easily performed by non-highly trained personnel, overcoming the major bottleneck of NGS analyses. Finally, the economic impact on testing laboratories was also taken into consideration, as the usage of the Flongle adaptor, along with the multiplexing capacity of the method and removal of the WGA step, allowed to greatly reduce the cost per assay (if desired the 20 min qPCR allows to further reduce the cost and removes the need to sequence all the samples). It is envisioned that this method will set the basis for additional developments of NGS-based methods, which could be easily implementable in routine testing laboratories, as the procedure detailed in this manuscript is highly adaptable to the specific needs of specific laboratories.

## Data availability statement

The original contributions presented in the study are included in the article/Supplementary material, further inquiries can be directed to the corresponding author.

## Author contributions

SA, FR, and AC-R performed the analyses and revised the manuscript. MP was responsible for funding acquisition, revision, and editing of the manuscript. AG-M envisioned the study, analyzed the data, and wrote the first draft of the manuscript. All authors contributed to the article and approved the submitted version.

## Funding

This work was financially supported by the Seafood Age Project, which was co-financed by the Interreg Atlantic Area Program (EAPA_758/2018) through the European Development Fund (ERDF). SA was financed by a Ph.D. grant from the Fundação para a Ciência e a Tecnologia (SFRH/BD/140396/2018). AG-M acknowledges funding from the Fundação para a Ciência e a Tecnologia through the Scientific Employment Stimulus Program (2021.02810.CEECIND).

## Conflict of interest

The authors declare that the research was conducted in the absence of any commercial or financial relationships that could be construed as a potential conflict of interest.

## Publisher's note

All claims expressed in this article are solely those of the authors and do not necessarily represent those of their affiliated organizations, or those of the publisher, the editors and the reviewers. Any product that may be evaluated in this article, or claim that may be made by its manufacturer, is not guaranteed or endorsed by the publisher.
