# Supplementary material for: Application of MinION sequencing as a tool for the rapid detection and characterization of Listeria monocytogenes in smoked salmon
Source: Front Microbiol. 2022 Aug 10;13:931810. doi: 10.3389/fmicb.2022.931810 (PMC9399719; doi:10.3389/fmicb.2022.931810)
Supplement: Supplementary file 1 [file Data_Sheet_1.docx]

**Supporting information**

**Long-read MinION detection and characterization of *Listeria monocytogenes* in smoked salmon**

Sarah Azinheiro^1, 2^, Foteini Roumani^1, 2^, Ana Costa-Ribeiro^1, 3^, Marta Prado^1^, Alejandro Garrido-Maestu^1*^

^1^Food Quality & Safety Research Group, International Iberian Nanotechnology Laboratory, Av. Mestre José Veiga s/n, 4715-330 Braga, Portugal

^2^Department of Analytical Chemistry, Nutrition and Food Science, Faculty of Veterinary Science, University of Santiago de Compostela, 27002 Lugo, Spain

^3^Departament of Biochemistry, Genetics and Immunology, University of Vigo, 36310 Vigo, Spain

Tel: 351 253 140 112– 2236

Fax: 351 253 140 119

*Corresponding author Alejandro Garrido-Maestu

E-mail: [alejandro.garrido@inl.int](mailto:alejandro.garrido@inl.int)

**1. Magnetic beads functionalization**

The functionalization protocol was previously reported by Garrido-Maestu et al., (Garrido-Maestu et al., 2020). Magnetic Nanospheres (MNP) from AbraMag^®^ with an average size of 500 nm were acquired. To assure proper orientation of the Ab, the MNP were acquired coated with protein A. These particles were purchased from Abraxis Inc. (Warminster, PA, USA). The MNPs were washed twice with 1 mL of 0.1 M sodium phosphate buffer with Tween^®^20 (PBT, 19 mM NaH_2_PO_4_, 81 mM Na_2_HPO_4_, 0.05 % Tween^®^20, pH 7.4). The recovery of the MNP was performed in a magnetic particle concentrator (Dynal® MPC (Invitrogen, Carlsbad, CA, USA)) for 2 min. After the initial washing steps, 60 µg/mL of Ab were added to the MNPs, in a final volume 10 times higher, to allow the distribution of the Ab in the particles. The solution was incubated for 1 h at room temperature in a Mini Tube Rotator (Fisher Scientific) set at 10 rpm. Finally, the MNPs were washed again twice as described above.

**2. Serogroup amplification plots**

Figure S1. Serogroup IV, strain WDCM 00021, ORF2819+ and ORF2110+.


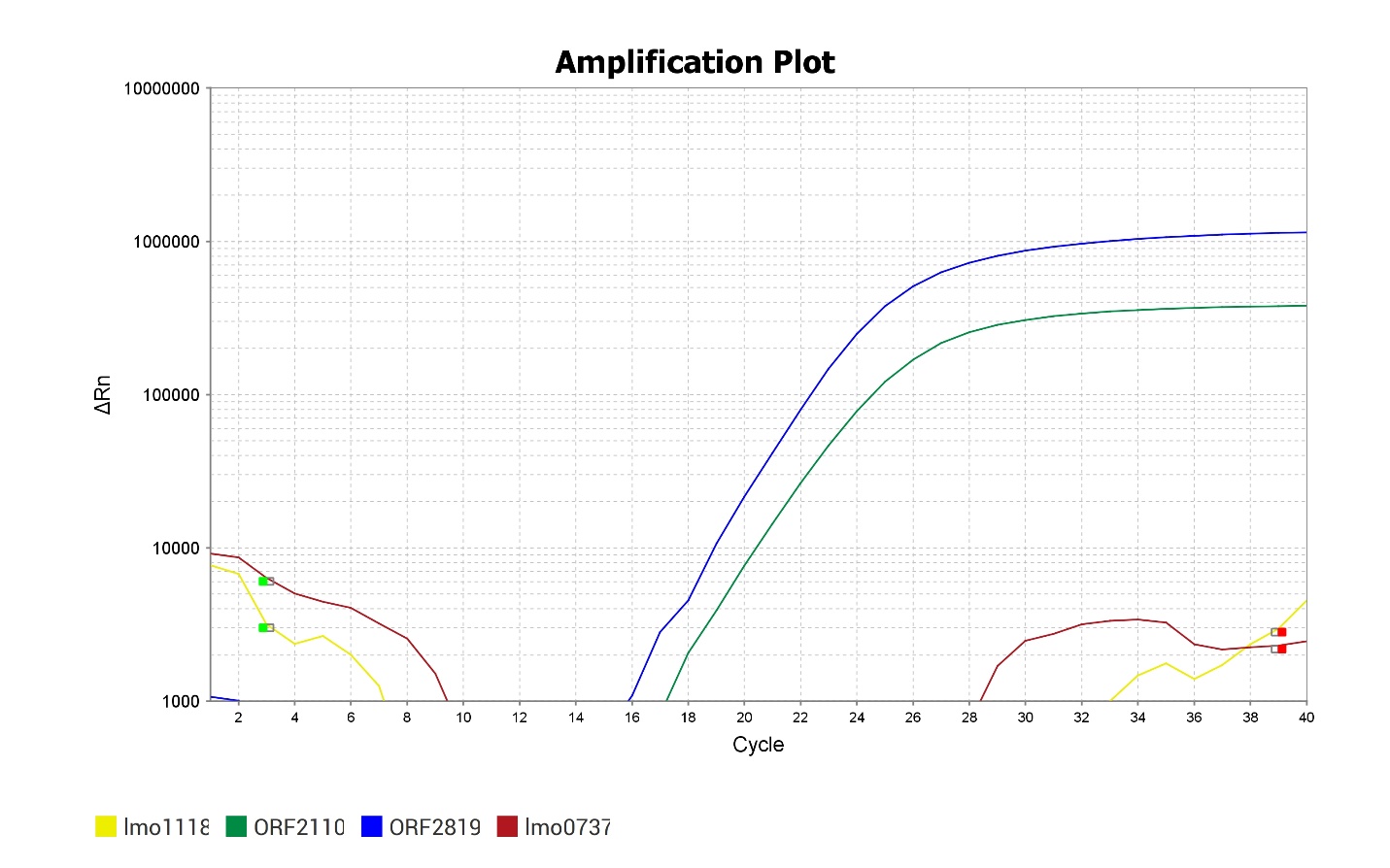


Figure S2. Serogroup III, strains from mollusk from Spain (a) and chicken from Portugal (b), ORF2819+.


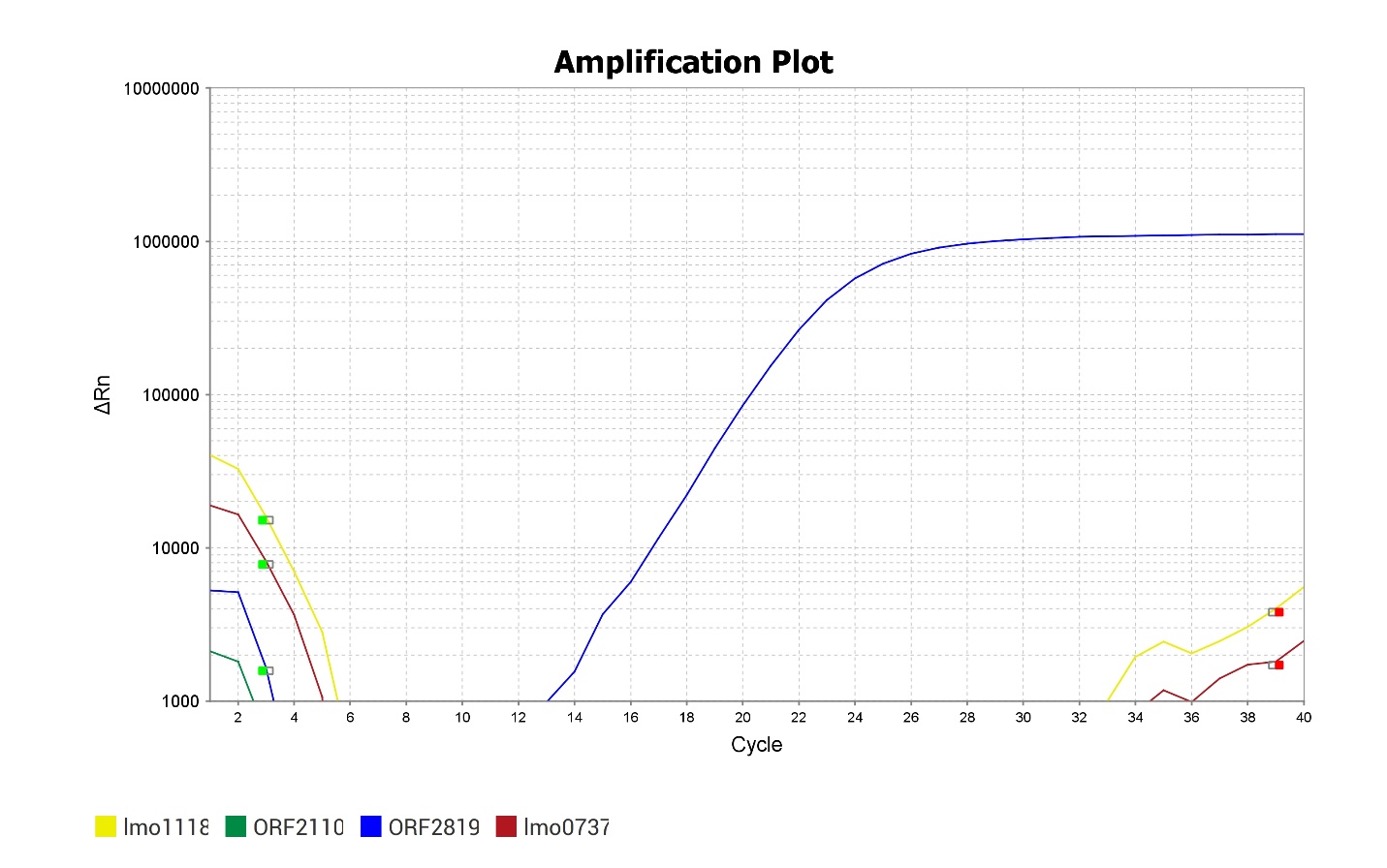


a)


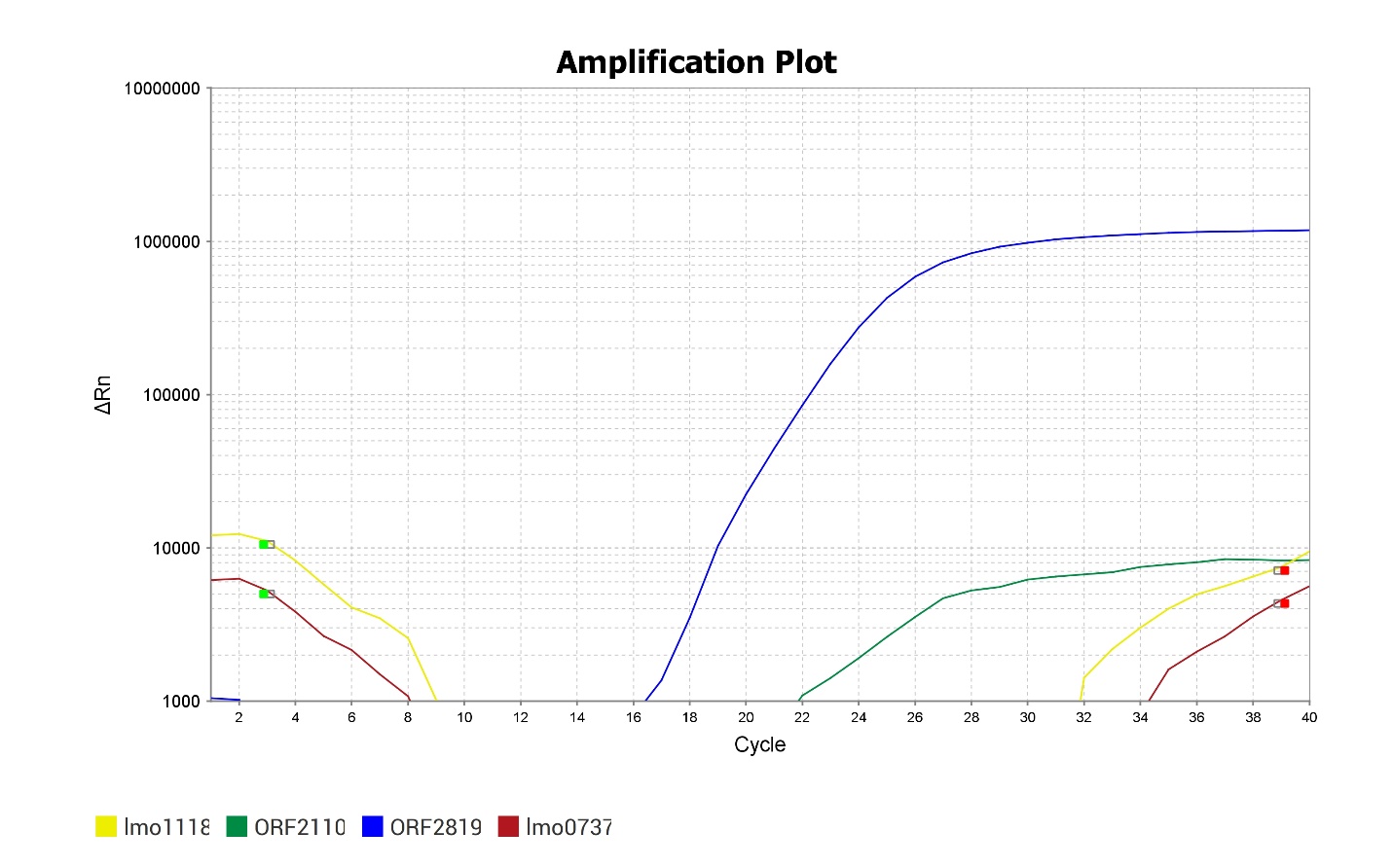


b)

Figure S3. Serogroup I, strains from chestnuts (a) and chicken from Spain (b), *lmo0737*+.


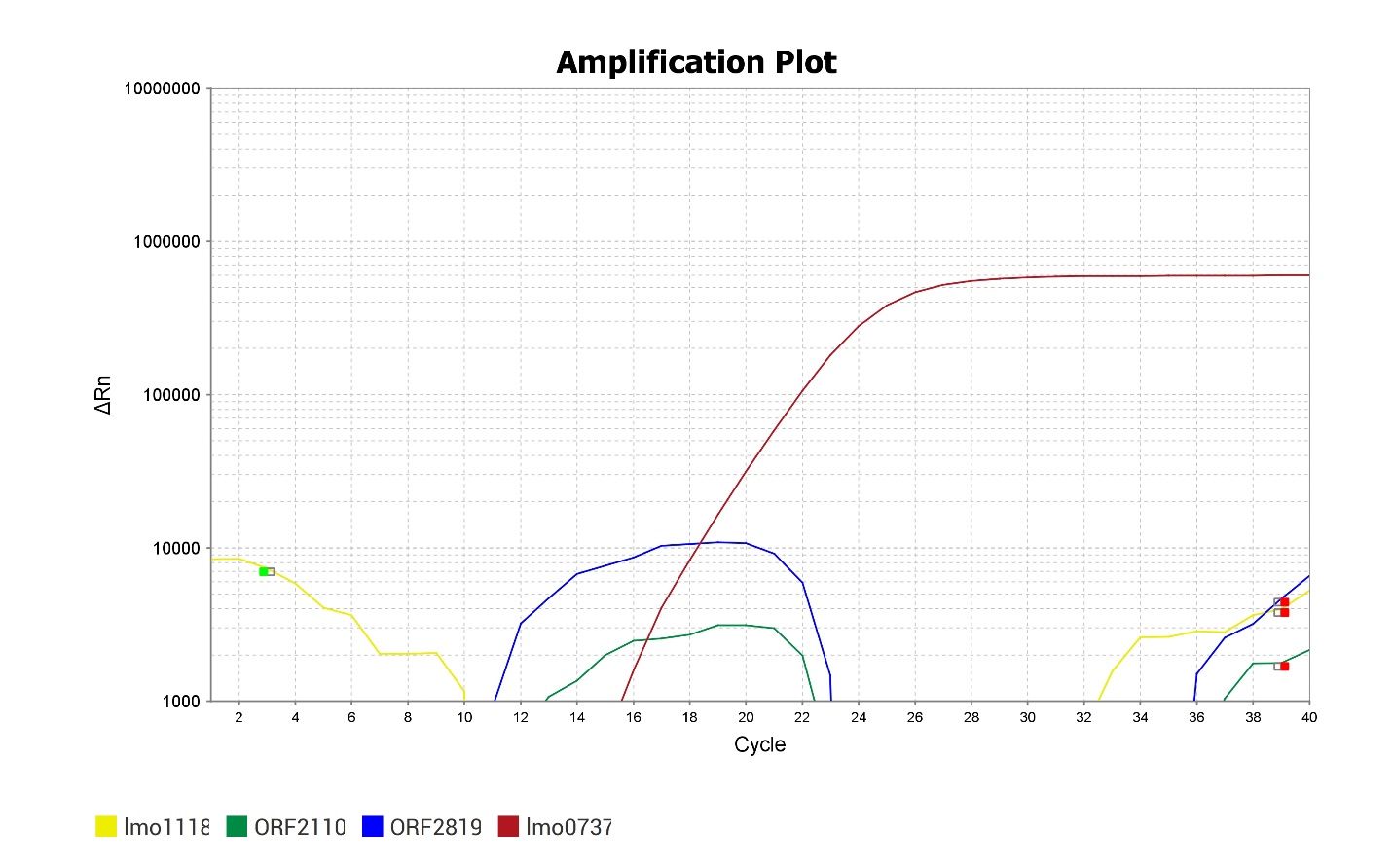


a)


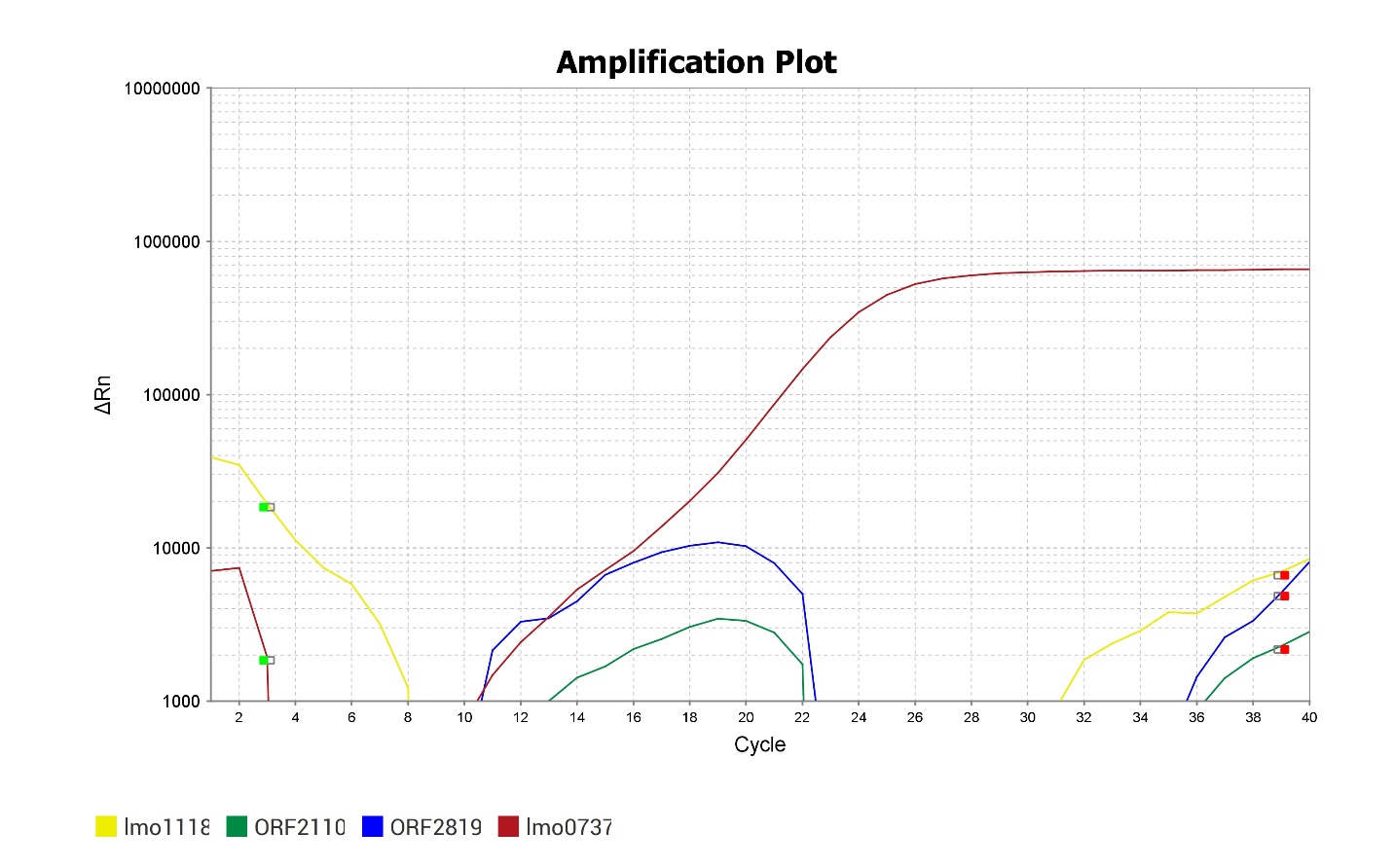


b)
